# Smartphone-Derived Movement Analysis for Musculoskeletal Assessment: Smartphone-Estimated Relative Vertical Power During the Sit-to-Stand Test as an Accessible Predictor of Knee Extensor Strength in Older Adults

**DOI:** 10.3390/medicina62061195

**Published:** 2026-06-21

**Authors:** Chanon Fapinyo, Weerasak Tapanya, Nitiphoom Sinnathakorn, Pasa Sukson, Warunyou Ngiamphaisan, Noppharath Sangkarit

**Affiliations:** 1 Department of Orthopaedics, School of Medicine, University of Phayao, Phayao 56000, Thailand; chanon.fa@up.ac.th (C.F.); nitiphoom.si@up.ac.th (N.S.); 2 Department of Physical Therapy, School of Allied Health Sciences, University of Phayao, Phayao 56000, Thailand; noppharath.sa@up.ac.th; 3 Department of Medicine, School of Medicine, University of Phayao, Phayao 56000, Thailand; pasa.su@up.ac.th; 4 Department of Anesthesiology, School of Medicine, University of Phayao, Phayao 56000, Thailand; warunyou.ng@up.ac.th

**Keywords:** sarcopenia, sit-to-stand test, muscle power, biomechanics, smartphone application

## Abstract

*Background and Objectives:* Assessing knee extensor (KE) strength is important for detecting muscle weakness in older adults, yet dynamometry is often impractical in community settings. This study examined whether smartphone-derived kinematics during the Five Times Sit-to-Stand Test (FTSST) could predict seated isometric KE strength. *Materials and Methods:* A cross-sectional study included 105 community-dwelling older adults (68.19 ± 5.85 years). A smartphone application extracted rising time, vertical velocity, and smartphone-estimated relative vertical power during the FTSST. KE strength was measured as maximum voluntary isometric contraction (MVIC) using fixed-frame dynamometry with a Lafayette dynamometer head. Bioelectrical impedance-derived body composition variables were reported descriptively but excluded from the primary prediction models to maintain a transparent movement-based model independent of device-specific body-composition estimates. Hierarchical regression models used smartphone-derived variables and transparent non-BIA covariates. Agreement was examined using Bland–Altman analysis. *Results:* Smartphone-estimated relative vertical power showed the strongest correlation with MVIC (r = 0.787, *p* < 0.001). The combined model including sex, age, femur length, and smartphone-estimated relative vertical power explained 71.6% of MVIC variance (adjusted R^2^ = 0.716, SEE = 3.276 kg), outperforming vertical velocity, rising time, and total FTSST time models. Internal validation using repeated 10-fold cross-validation showed CV-R^2^ = 0.701, CV-adjusted R^2^ = 0.689, CV-RMSE = 3.343 kg, and CV-MAE = 2.739 kg. Bland–Altman analysis showed minimal mean bias (0.00 kg), 95% limits of agreement from −6.296 to 6.296 kg, and significant proportional bias (slope = −0.172, *p* = 0.002), indicating overestimation in weaker individuals and underestimation in stronger individuals. *Conclusions:* Consistent with our hypothesis, smartphone-estimated relative vertical power was the strongest kinematic predictor of seated isometric KE strength among the evaluated FTSST-derived variables. This approach may support community screening and monitoring, but it should not replace standardized dynamometry for precise individual-level strength quantification.

## 1. Introduction

Aging is inherently associated with a progressive decline in skeletal muscle mass and neuromuscular function, a condition widely recognized as sarcopenia [1,2,3]. Among the physiological alterations observed in older adults, the deterioration of lower-limb muscle strength, particularly the knee extensor muscles, is a critical concern [4,5]. The knee extensors are essential for generating the torque required to perform fundamental activities of daily living, such as walking, stair climbing, and transitioning from a seated to a standing position [6,7]. Consequently, age-related deficits in knee extensor strength are strongly correlated with an increased risk of falls, a loss of functional independence, and elevated mortality rates in the geriatric population [8,9]. Accurate assessment of lower-limb muscle strength is therefore vital for early clinical intervention and the continual monitoring of rehabilitation progress.

In clinical and biomechanical research, maximum voluntary isometric contraction (MVIC), measured via hand-held, fixed-frame, or isokinetic dynamometry, is widely used for quantifying isolated muscle strength [10]. However, the routine implementation of these measurement techniques in community-based or primary care settings is constrained by the need for equipment, standardized stabilization procedures, calibration, and trained personnel to ensure measurement validity and reliability [9,11]. To overcome these logistical barriers, clinicians frequently rely on functional field tests as practical surrogate measures of lower-limb capability.

The Five Times Sit-to-Stand Test (FTSST) is one of the most widely adopted clinical tools for evaluating functional lower-limb strength and dynamic balance [5,12]. Biomechanically, the sit-to-stand maneuver is a demanding weight-bearing task. It requires the neuromuscular system to generate adequate lower-extremity joint moments to vertically accelerate the body center of mass against gravity [13,14]. Traditionally, the FTSST is evaluated solely based on the total time required to complete five consecutive cycles [5,12]. While total completion time provides a general overview of functional mobility, it lacks biomechanical specificity. The cumulative time encompasses the concentric rising phase, the eccentric sitting phase, and the transition periods between movements. This aggregation may obscure the rate of force development and upward propulsion generated during the critical rising phase [13,15]. Furthermore, temporal metrics alone cannot account for inter-individual variations in body mass and segment lengths, which influence the mechanical demand required to complete the task.

From a human movement science perspective, muscle power is a sensitive indicator of age-related functional decline [16,17]. Physiological aging is characterized by preferential decline in type II fast-twitch muscle fiber function, which reduces the capacity to execute rapid movements [18,19]. During the concentric rising phase of the sit-to-stand task, rapid motor-unit recruitment and force development are required to generate sufficient vertical acceleration. The lower-limb extensors must generate force to raise the body center of mass from sitting to standing [14,20]. By using kinematic analysis to isolate this upward phase, variables such as rising time, vertical velocity, and app-derived power estimates can be extracted. In the present manuscript, however, the app-derived power variable is consistently described as smartphone-estimated relative vertical power because it is calculated from an empirically calibrated regression equation rather than directly measured mechanical power.

Recent advancements in mobile health technology have enabled the extraction of high-resolution kinematic parameters using accessible smartphone-based video analysis applications [21,22]. These tools can track spatial and temporal movement characteristics and support estimation of sit-to-stand performance under field conditions [21,22].

Previous studies have validated smartphone-based sit-to-stand applications against force-plate-derived power and have supported their use for estimating task-level sit-to-stand performance [21,23]. However, validation against force-plate-derived sit-to-stand power addresses a different methodological question from whether smartphone-derived FTSST variables can estimate an external clinical criterion of isolated lower-limb force capacity, such as seated isometric knee extensor MVIC. In addition, the comparative predictive value of total FTSST time, app-derived rising time, vertical velocity, and smartphone-estimated relative vertical power has not been clearly established within the same analytical framework in community-dwelling Thai older adults.

Therefore, the present study examined the relationships between FTSST-derived performance variables and seated isometric knee extensor MVIC. The primary objective was to compare multiple regression models based on total FTSST time, rising time, vertical velocity, and smartphone-estimated relative vertical power, while adjusting for transparent non-BIA covariates. Based on the biomechanical role of lower-limb extensor force generation during the rising phase of sit-to-stand, we hypothesized that smartphone-estimated relative vertical power would demonstrate the strongest association with knee extensor MVIC and would show the best model performance among the evaluated FTSST-derived variables.

Establishing an accurate and biomechanically interpretable prediction model for knee extensor strength holds clinical significance. It facilitates the translation of movement-based assessment into community settings. By utilizing accessible smartphone technology to extract specific kinematic variables, healthcare professionals may identify individuals who require confirmatory strength assessment without immediate reliance on specialized dynamometry. This approach may enhance screening for muscle weakness, support fall-risk stratification, and guide targeted resistance training or rehabilitation programs in older adults.

## 2. Materials and Methods

### 2.1. Study Design and Participants

This study adopted a cross-sectional analytical design employing a correlational approach to explore the predictive relationships between kinematic performance variables during the FTSST and knee extensor muscle strength. The core objective was to develop and compare multiple regression models using smartphone-derived parameters, specifically total FTSST time, rising time, vertical velocity, and smartphone-estimated relative vertical power, derived from a validated video analysis application.

Sample size estimation was determined using G*Power software (version 3.1.9.6; Heinrich Heine University Düsseldorf, Düsseldorf, Germany). Based on an a priori power analysis for a bivariate normal model, assuming a minimum expected correlation coefficient of r = 0.30, α = 0.05, and power = 0.90, a minimum of 88 participants was required [24]. Participants were recruited using convenience sampling in collaboration with local community leaders and healthcare volunteers in the Muang Phayao district, Thailand. Convenience sampling was used because the study was conducted in a community-based setting and required identification of older adults who could safely complete the physical assessments, including seated knee extensor MVIC testing and the FTSST. This sampling strategy was practical for evaluating the feasibility and predictive performance of a smartphone-based assessment under real-world community conditions.

A total of 125 community-dwelling older adults aged ≥60 years were initially screened. Inclusion criteria encompassed older adults capable of independent ambulation without assistive devices and possessing stable general health. Individuals were excluded if they presented with lower-limb musculoskeletal pathologies, such as symptomatic osteoarthritis or rheumatoid arthritis, a history of lower-extremity surgery or fracture, neurological disorders affecting motor control, significant sensory impairments, or recent use of medications with cognitive side effects [5,24,25]. Following screening, 20 individuals were excluded: 5 required walking aids, 10 had restricted joint mobility due to osteoarthritis, and 5 had a history of total knee arthroplasty. The final cohort included 105 participants. The demographic and functional characteristics of the participants are presented in Table 1.

The study protocol was approved by the Human Research Ethics Committee of the University of Phayao (Approval No. HREC-UP-HSST 1.2/065/68) and adhered to the ethical standards of the Declaration of Helsinki. Written informed consent was obtained from all participants prior to data collection.

### 2.2. Anthropometric Assessments and Bioelectrical Impedance-Derived Body Composition Assessments

Following the consent process, baseline demographic data and directly measured anthropometric variables were recorded, including body height, body weight, body mass index (BMI), femur length, and calf circumference. BMI was calculated as body mass divided by height squared (kg/m^2^). Calf circumference was recorded as a simple directly measured anthropometric descriptor and was examined in exploratory correlation analysis.

Bioelectrical impedance-derived body composition variables were also recorded descriptively using a Tanita BC-730 bioelectrical impedance analyzer (Tanita Corporation, Tokyo, Japan). The recorded BIA-derived variables included body fat percentage, bone mass, skeletal muscle mass, and skeletal muscle mass index (SMI). SMI was calculated as skeletal muscle mass divided by height squared (kg/m^2^). These variables were included in Table 1 to provide a more complete descriptive profile of participant body composition. However, because BIA-derived values depend on device-specific estimation algorithms, they were not included in the primary prediction models. The primary regression analyses therefore remained focused on smartphone-derived kinematic variables and transparent non-BIA covariates.

Prior to the formal physical assessments, participants underwent a three-minute familiarization session. The primary assessments consisted of the MVIC of the knee extensor muscles and the FTSST. To control for potential order effects and minimize localized fatigue, the sequence of these two tests was randomized, with a mandatory five-minute rest period administered between tests.

### 2.3. Assessment of Knee Extensor Muscle Strength (MVIC)

The isometric strength of the knee extensor muscles was quantified using a standardized MVIC protocol. Participants were seated on an adjustable NK testing table, configured to individual segment lengths to ensure mechanical alignment. The knee joint was positioned at 120° on the anatomical joint-angle scale, where 180° represents full knee extension; this corresponds to approximately 60° of knee flexion. The position was confirmed using a universal goniometer. This standardized posture was used to provide a reproducible and clinically tolerable testing position for older adults.

**Figure 1:**
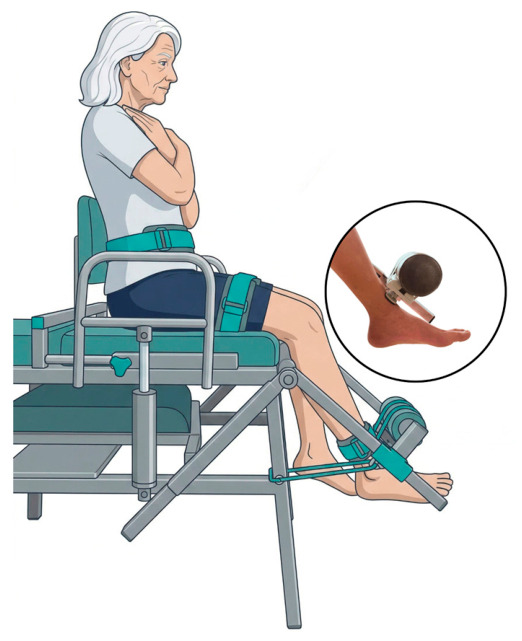
Setup and execution of the knee extensor MVIC test using fixed-frame dynamometry with a Lafayette dynamometer head rigidly mounted to the NK table extension.

To isolate quadriceps activation and restrict compensatory trunk or pelvic movements, participants were securely stabilized using adjustable non-slip straps. Knee extensor MVIC was measured using fixed-frame dynamometry with a Lafayette dynamometer head (Lafayette Instrument Company, Lafayette, IN, USA) rigidly mounted to the NK table extension. This fixed-frame configuration was used to minimize examiner-strength dependence and improve the mechanical consistency of force measurement. The dynamometer pad was positioned perpendicular to the anterior surface of the lower leg, 1 cm proximal to the lateral malleolus (Figure 1). Following a visual demonstration, participants were instructed to exert maximal forward force against the dynamometer for a sustained four-second contraction while avoiding breath-holding or the Valsalva maneuver. Three separate trials were executed, separated by a two-minute passive recovery interval. The highest peak force value (kg) across the three trials was retained for statistical analysis [5,24,25].

### 2.4. Five Times Sit-to-Stand Test (FTSST) and Video-Based Kinematic Analysis

The FTSST was administered to evaluate dynamic lower-limb performance. Participants were seated barefoot on a stable armless chair with non-slip, non-wheeled legs, with the seat height adjusted to establish approximately 90° of flexion at the hip, knee, and ankle joints. Chair height was individually adjusted to achieve approximately 90° of hip, knee, and ankle flexion at the starting seated position. This standardization was used to reduce variation in initial lower-limb joint configuration across participants. The upper limbs were crossed firmly across the chest. Upon the command “Start,” participants completed five consecutive sit-to-stand cycles at maximal safe speed, ensuring full hip and knee extension during the standing phase and complete seat contact during the sitting phase. The mean duration of three valid trials, separated by two-minute rest intervals, was recorded.

**Figure 2:**
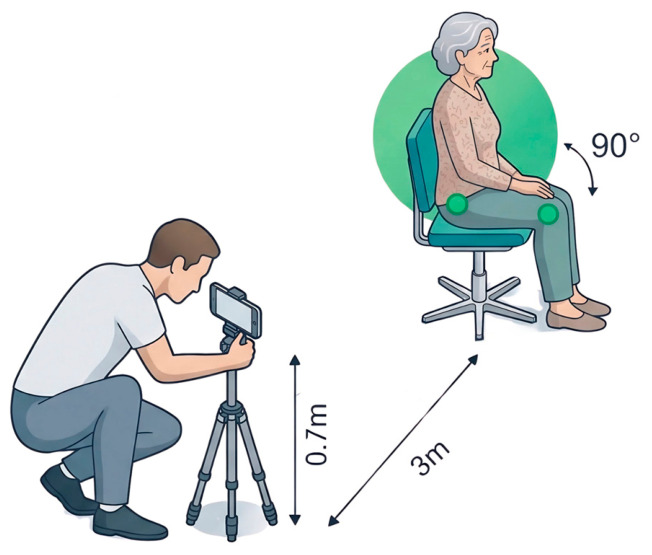
Smartphone-based setup for extracting FTSST-derived kinematic variables during the sit-to-stand task. The smartphone was mounted at a fixed height of 0.7 m and positioned 3 m lateral to the participant, following the validated setup described by Ruiz-Cárdenas et al. [21,23].

Simultaneously, kinematic variables during the rising phase of the third repetition were quantified using a validated Sit-to-Stand smartphone application (version 2.0.2; Juan Diego Ruiz-Cárdenas, Murcia, Spain), installed on an iPhone 14 Pro (Apple Inc., Cupertino, CA, USA) at 240 frames/s. The device was mounted horizontally on a tripod at a height of 0.7 m and positioned 3 m lateral to the participant, following the validated setup described by Ruiz-Cárdenas et al. [21,23] (Figure 2). High-speed video was captured to ensure precise temporal resolution. A high-contrast visual marker was affixed to the superior aspect of the greater trochanter to track movement during the rising phase.

The third repetition was selected for kinematic analysis because it represents a mid-task cycle after the initial start-up movement and before potential fatigue, slowing, or altered movement strategy during the final repetitions. This approach was intended to capture a relatively stable rising strategy within the five-repetition sequence.

The analytical framework isolated the concentric rising phase, defined temporally from the initiation of anterior pelvic displacement to the attainment of peak vertical height at full extension. This interval was automatically computed as rising time (t). In the validated app-derived equation, vertical displacement (d) was represented using a femur-length-based anthropometric proxy. This value should not be interpreted as the actual measured vertical excursion of the greater trochanter during sit-to-stand. Consequently, mean vertical velocity was calculated as:

v=d/t

where v is vertical velocity in m/s, d is estimated vertical displacement in meters, and t is rising time in seconds.

Smartphone-estimated relative vertical power was estimated using the validated regression algorithm reported by Ruiz-Cárdenas et al. [21,23]:

(1) P=2.773−(6.228×t)+(18.224×d)

where P is smartphone-estimated relative vertical power in W/kg, t is rising time in seconds, and d is estimated vertical displacement in meters.

The equation was originally developed by Ruiz-Cárdenas et al. using force-plate-derived sit-to-stand power data from 48 healthy individuals aged 26–81 years [23]. Therefore, this variable should be interpreted as a regression-derived estimate of relative sit-to-stand power rather than a direct measurement of mechanical power, whole-body mechanical work, ground reaction force, or joint-level muscle power. The coefficients in the equation represent empirical calibration coefficients from the original validation work and should not be interpreted as being derived directly from Newtonian force–acceleration calculation or inverse dynamics.

Although the smartphone model, app version, camera frame rate, camera configuration, and published power equation were reported, any additional internal app-processing steps beyond the published equation may depend on the application implementation. Video analysis was conducted by a trained assessor who was blinded to the MVIC results.

### 2.5. Statistical Analysis

All statistical analyses were conducted using IBM SPSS Statistics (version 26.0; IBM Corp., Armonk, NY, USA), with the alpha level set at 0.05. Normality was assessed using the Shapiro–Wilk test and visual inspection of histograms, Q–Q plots, and scatter plots. Pearson correlation coefficients were used for the main bivariate analyses to maintain comparability with the linear regression framework. When variables deviated from normality or showed visible heterogeneous spread, Spearman rank correlations were calculated as sensitivity analyses.

Descriptive statistics were used to summarize demographic, anthropometric, functional, and kinematic variables. Because the sample was sex-imbalanced, sex-stratified descriptive statistics were reported, and a sex × smartphone-estimated relative vertical power interaction term was added to the final model to explore whether the association between smartphone-estimated relative vertical power and MVIC differed by sex.

Because multiple bivariate correlations were examined, correlation analyses were interpreted as exploratory and descriptive. Confirmatory inference was focused on the prespecified primary hypothesis and the adjusted regression models. Benjamini–Hochberg false-discovery rate correction was applied to the exploratory correlation tests reported in Table 2.

To address methodological concerns regarding device-derived body composition estimates, all bioelectrical impedance-derived variables, including body fat percentage, bone mass, and skeletal muscle mass, were excluded from the main predictive analyses. Multiple regression analyses were conducted using smartphone-derived kinematic variables and non-BIA covariates only. The dependent variable was knee extensor MVIC. The primary kinematic predictors were total FTSST time, rising time, vertical velocity, and smartphone-estimated relative vertical power. Age, sex, and femur length were included as non-BIA covariates because they represent demographic and directly measured anthropometric characteristics relevant to lower-limb function and sit-to-stand mechanics.

Body weight and calf circumference were considered in sensitivity analyses because they showed weak exploratory correlations with MVIC. However, they were not included in the primary regression models because the primary objective was to test the incremental value of smartphone-derived movement variables beyond sex, age, and femur length, while avoiding unnecessary model complexity in a sample of 105 participants. A sensitivity model was therefore fitted by adding body weight and calf circumference to the final power-based model.

Model comparison was performed using adjusted R^2^, standard error of the estimate (SEE), Akaike information criterion (AIC), and Bayesian information criterion (BIC). For nested model comparisons, ΔR^2^ and partial F-tests were used. Because the models containing different FTSST-derived kinematic predictors were not nested, direct partial F-tests between these candidate models were not performed; instead, AIC, BIC, adjusted R^2^, and SEE were used to compare relative model fit. Ninety-five percent confidence intervals were reported for correlation coefficients and unstandardized regression coefficients.

The incremental value of smartphone-estimated relative vertical power beyond the demographics-only baseline model was quantified using ΔR^2^, partial R^2^, and partial F-testing. Internal validation of the final power-based model was performed using repeated 10-fold cross-validation with 100 repetitions, consistent with transparent reporting principles for prediction-model studies [26]. Cross-validated R^2^, cross-validated adjusted R^2^, root mean square error (RMSE) and mean absolute error (MAE) were calculated from out-of-fold predictions and compared with the apparent in-sample performance.

Multicollinearity was monitored using the variance inflation factor (VIF), with values greater than 10 considered indicative of problematic collinearity. Agreement between measured knee extensor MVIC and model-estimated MVIC from the final power-based model was further examined using Bland–Altman analysis [27]. The mean bias was calculated as the average difference between estimated and measured MVIC values, and the 95% limits of agreement were calculated as the mean bias ± 1.96 standard deviations of the differences. A proportional-bias test was conducted by regressing the difference between estimated and measured MVIC on the mean of measured and estimated MVIC. MAE, RMSE, and influence diagnostics including Cook’s distance, standardized residuals, and leverage values were also examined.

## 3. Results

### 3.1. Demographic Characteristics, Body Composition, and Functional Performance

A total of 105 community-dwelling older adults, comprising 25 males (23.8%) and 80 females (76.2%), participated in this study. The mean age was 68.19 ± 5.85 years. The mean body weight, height, and BMI were 53.39 ± 11.14 kg, 153.64 ± 8.98 cm, and 22.63 ± 4.42 kg/m^2^, respectively. Directly measured anthropometric variables included femur length and calf circumference. Bioelectrical impedance-derived descriptive variables showed mean body fat percentage of 28.25 ± 11.99%, bone mass of 2.05 ± 0.42 kg, skeletal muscle mass of 36.02 ± 6.71 kg, and SMI of 15.20 ± 2.01 kg/m^2^.

Regarding functional performance, the average FTSST completion time was 11.94 ± 2.93 s. Smartphone-derived kinematic analysis of the rising phase showed a mean rising time of 0.95 ± 0.23 s, vertical velocity of 0.38 ± 0.09 m/s, and smartphone-estimated relative vertical power of 3.13 ± 1.24 W/kg. The mean MVIC of knee extensor strength was 22.78 ± 6.15 kg.

Because the sample included more women than men, sex-stratified descriptive statistics are presented in Table 1. Women had higher mean body fat percentage than men (31.12 ± 11.69% vs. 19.05 ± 7.60%), whereas men had higher skeletal muscle mass (43.64 ± 7.90 kg vs. 33.63 ± 4.02 kg), SMI (16.23 ± 2.87 kg/m^2^ vs. 14.88 ± 1.53 kg/m^2^), smartphone-estimated relative vertical power (4.17 ± 1.00 W/kg vs. 2.81 ± 1.13 W/kg), and MVIC (29.06 ± 4.69 kg vs. 20.81 ± 5.16 kg). These descriptive differences support the inclusion of sex as a covariate in the regression models. Descriptive characteristics are presented in Table 1.

### 3.2. Correlation Analysis

Shapiro–Wilk testing indicated that MVIC (W = 0.995, *p* = 0.971), vertical velocity (W = 0.978, *p* = 0.079), and smartphone-estimated relative vertical power (W = 0.980, *p* = 0.119) did not significantly deviate from normality. In contrast, age (W = 0.937, *p* < 0.001), femur length (W = 0.934, *p* < 0.001), rising time (W = 0.900, *p* < 0.001), and total FTSST time (W = 0.910, *p* < 0.001) deviated from normality. Therefore, Spearman rank correlations were calculated as sensitivity analyses for variables with non-normal distributions or visually heterogeneous spread.

#### 3.2.1. Correlation Between Knee Extensor Strength and Participant Characteristics

Pearson correlation analysis demonstrated significant associations between knee extensor MVIC and several participant characteristics (Table 2). Sex showed a moderate-to-strong positive association with MVIC (r = 0.574, 95% CI = 0.430 to 0.690, *p* < 0.001), whereas age showed a moderate negative association (r = −0.495, 95% CI = −0.627 to −0.335, *p* < 0.001). Among directly measured anthropometric variables, height (r = 0.502, 95% CI = 0.343 to 0.633, *p* < 0.001) and femur length (r = 0.451, 95% CI = 0.284 to 0.592, *p* < 0.001) were positively correlated with MVIC, while BMI showed no significant association (r = −0.025, 95% CI = −0.216 to 0.167, *p* = 0.797).

Body weight showed a weak positive correlation with MVIC (r = 0.249, 95% CI = 0.060 to 0.421, *p* = 0.010), and calf circumference also showed a weak positive correlation with MVIC (r = 0.208, 95% CI = 0.017 to 0.384, *p* = 0.033). These correlations were statistically significant but smaller than those observed for sex, age, femur length, and smartphone-estimated relative vertical power.

#### 3.2.2. Correlation Between Knee Extensor Strength and Smartphone-Derived Kinematic Parameters

**Figure 3:**
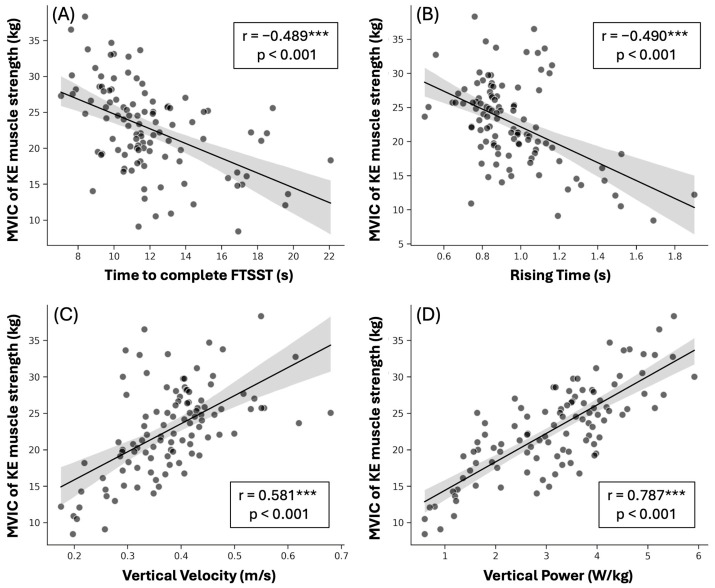
Scatter plots showing the relationships between knee extensor MVIC and FTSST-derived variables. (**A**) total FTSST time, (**B**) rising time, (**C**) vertical velocity, and (**D**) smartphone-estimated relative vertical power. The solid lines represent linear regression fits. *** indicates *p* < 0.001. Because the rising-time panel showed visually heterogeneous spread, Spearman rank correlations were also calculated as sensitivity analyses and are reported in Table 2. The grey shaded areas around the regression lines represent the 95% confidence intervals.

When evaluating functional performance, knee extensor MVIC showed a moderate negative correlation with traditional FTSST completion time (r = −0.489, 95% CI = −0.622 to −0.328, *p* < 0.001) and app-derived rising time (r = −0.490, 95% CI = −0.623 to −0.329, *p* < 0.001). Vertical velocity demonstrated a significant positive correlation with knee extensor MVIC (r = 0.581, 95% CI = 0.438 to 0.695, *p* < 0.001). Smartphone-estimated relative vertical power demonstrated the strongest correlation with knee extensor MVIC among the assessed FTSST-derived variables (r = 0.787, 95% CI = 0.701 to 0.850, *p* < 0.001) (Table 2; Figure 3).

Spearman sensitivity analyses produced directions and statistical significance consistent with the Pearson results. For FTSST-derived variables, Spearman correlations with MVIC were −0.513 for total FTSST time, −0.421 for rising time, 0.586 for vertical velocity, and 0.749 for smartphone-estimated relative vertical power, all *p* < 0.001. These findings support the robustness of the main correlation pattern, although the rising-time association was weaker when rank-based correlation was used.

The exploratory bivariate correlations between MVIC and the candidate variables remained statistically significant after Benjamini–Hochberg false-discovery rate correction. The overall interpretation was unchanged, with smartphone-estimated relative vertical power showing the strongest association with MVIC among the FTSST-derived variables.

#### 3.2.3. Multiple Linear Regression Analysis After Excluding Bioelectrical Impedance-Derived Variables

After excluding all bioelectrical impedance-derived body composition variables, regression analyses continued to support the primary biomechanical interpretation of the study. Across all models, age, sex, and femur length were entered as non-BIA covariates. The power-based model demonstrated the highest predictive performance for knee extensor MVIC, explaining 71.6% of the variance (adjusted R^2^ = 0.716, SEE = 3.276 kg, *p* < 0.001). In this model, smartphone-estimated relative vertical power remained a significant independent predictor of MVIC (B = 2.423, 95% CI = 1.717 to 3.129, β = 0.488, *p* < 0.001).

The velocity-based model explained 65.8% of the variance (adjusted R^2^ = 0.658, SEE = 3.596 kg), while the rising-time model explained 63.1% of the variance (adjusted R^2^ = 0.631, SEE = 3.732 kg). The traditional FTSST total-time model explained 62.3% of the variance (adjusted R^2^ = 0.623, SEE = 3.774 kg). These results indicate that the predictive value of smartphone-estimated relative vertical power was not dependent on device-specific body composition estimates but was primarily driven by the movement information captured during the rising phase of the FTSST (Table 3).

The power-based model using smartphone-estimated relative vertical power showed the most favorable model fit among the four candidate kinematic models, with the highest adjusted R^2^ and the lowest information criteria (adjusted R^2^ = 0.716; SEE = 3.276 kg; AIC = 254.052; BIC = 267.322). The velocity-based model showed lower explanatory performance and higher information criteria (adjusted R^2^ = 0.658; SEE = 3.596 kg; AIC = 273.647; BIC = 286.916), followed by the rising-time model (adjusted R^2^ = 0.631; SEE = 3.732 kg; AIC = 281.433; BIC = 294.703) and the FTSST total-time model (adjusted R^2^ = 0.623; SEE = 3.774 kg; AIC = 283.803; BIC = 297.073). Because these four candidate models used different kinematic predictors, they were treated as non-nested models and compared using information criteria rather than direct partial F-tests (Table 4).

To quantify the incremental value of smartphone-estimated relative vertical power, the final power-based model was also compared with the demographics-only baseline model including sex, age, and femur length. The baseline model explained 60.0% of MVIC variance (R^2^ = 0.600; adjusted R^2^ = 0.588; SEE = 3.943 kg). Adding smartphone-estimated relative vertical power increased the explained variance to R^2^ = 0.727 (adjusted R^2^ = 0.716; SEE = 3.276 kg), corresponding to ΔR^2^ = 0.127 and partial R^2^ = 0.318. This improvement was statistically significant (partial F-change (1, 100) = 46.371, *p* < 0.001), indicating that smartphone-estimated relative vertical power contributed meaningful predictive information beyond sex, age, and femur length alone.

Internal validation of the final model using repeated 10-fold cross-validation showed CV-R^2^ = 0.701, CV-adjusted R^2^ = 0.689, CV-RMSE = 3.343 kg, and CV-MAE = 2.739 kg. These values were modestly lower than the apparent in-sample performance, indicating limited optimism and acceptable internal stability.

To examine whether the predictive association between smartphone-estimated relative vertical power and MVIC differed by sex, a sex × smartphone-estimated relative vertical power interaction term was added to the final model. The interaction was not significant (B = −0.145, 95% CI = −1.636 to 1.346, *p* = 0.847; ΔR^2^ = 0.0001; F-change = 0.037), suggesting no clear evidence that the association differed between women and men in this sample. However, this result should be interpreted cautiously because the male subgroup was small.

Although body weight and calf circumference showed weak positive exploratory correlations with MVIC, they were not included in the primary regression models to avoid unnecessary model complexity and to maintain a parsimonious movement-based prediction model. In the sensitivity model adding body weight and calf circumference to the final power-based model, smartphone-estimated relative vertical power remained strongly associated with MVIC (B = 2.437, 95% CI = 1.736 to 3.138, *p* < 0.001). Body weight (B = 0.035, 95% CI = −0.040 to 0.110, *p* = 0.360) and calf circumference (B = 0.085, 95% CI = −0.144 to 0.315, *p* = 0.463) were not independently associated with MVIC. The adjusted R^2^ changed only slightly from 0.716 in the primary model to 0.721 in the sensitivity model, indicating that the exclusion of body weight and calf circumference did not materially alter the main finding.

All VIF values ranged from 1.058 to 1.881 and were below the prespecified threshold of 10, indicating no problematic multicollinearity among predictors.

#### 3.2.4. Agreement Between Measured and Model-Estimated Knee Extensor Strength

Bland–Altman analysis was conducted to examine the agreement between measured MVIC and model-estimated MVIC derived from the final power-based model. The analysis showed a mean bias of 0.000 kg, with 95% limits of agreement ranging from −6.296 to 6.296 kg. A total of 3 participants (2.9%) fell outside the 95% limits of agreement, which is consistent with the expected proportion of approximately 5%. The mean absolute error was 2.610 kg, and the root mean square error was 3.197 kg.

**Figure 4:**
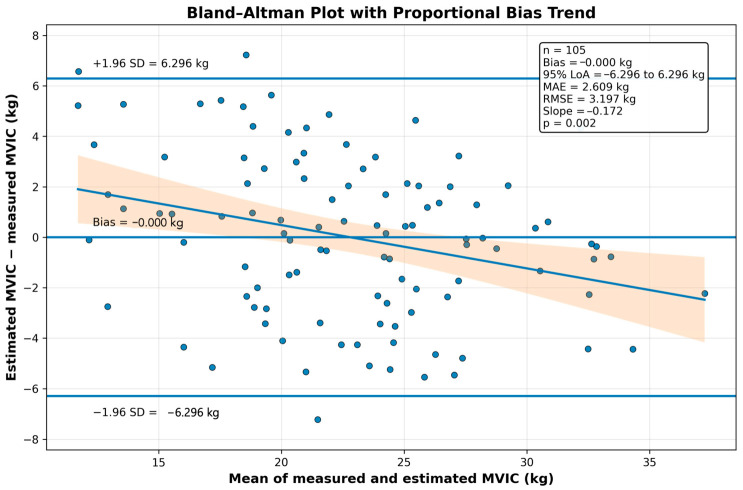
Bland–Altman plot showing agreement between measured knee extensor MVIC and model-estimated MVIC derived from the final power-based regression model. The horizontal solid line represents the mean bias, and the upper and lower horizontal lines represent the 95% limits of agreement. The oblique regression line and shaded confidence band indicate the proportional-bias trend. A total of 3 observations (2.9%) fell outside the 95% limits of agreement.

A proportional-bias test indicated a small but statistically significant association between the mean of measured and estimated MVIC and the prediction error (B = −0.172, 95% CI = −0.281 to −0.062, *p* = 0.002). This indicates that the model tended to overestimate MVIC in individuals with lower strength values and underestimate MVIC in individuals with higher strength values (Figure 4). The proportional-bias equation was BA_Diff = 3.913–0.172 × BA_Mean, where BA_Diff represents predicted minus measured MVIC and BA_Mean represents the mean of predicted and measured MVIC. Based on this equation, the model would be expected to overestimate MVIC by approximately 1.85 kg at a Bland–Altman mean of 12 kg and underestimate MVIC by approximately 2.11 kg at a Bland–Altman mean of 35 kg. Therefore, although the mean bias was negligible at the group level, the direction of proportional bias was clinically relevant for individual interpretation near low-strength thresholds.

The 95% limits of agreement of approximately ±6.3 kg indicate that the model is not sufficiently precise to replace dynamometry for individual-level strength quantification. However, the level of agreement may still be useful for broad community screening or longitudinal monitoring when interpreted as a risk-stratification measure rather than as a diagnostic measurement.

Influence diagnostics did not indicate severe influential cases. The maximum Cook’s distance was 0.068, and standardized residuals ranged from −2.208 to 2.205, indicating that no participant exceeded conventional thresholds for extreme residual influence.

## 4. Discussion

The present study investigated whether smartphone-derived kinematic variables extracted from the FTSST could predict seated isometric knee extensor MVIC in community-dwelling older adults. The primary finding was that smartphone-estimated relative vertical power was the strongest FTSST-derived predictor of seated isometric knee extensor MVIC among the evaluated variables. This finding supports the study hypothesis and indicates that the movement information captured during the rising phase of the FTSST provides clinically relevant information about lower-limb extensor force capacity.

The association observed in this study should be interpreted within the specific measurement context. MVIC was measured at a standardized knee joint angle of 120° on a 180° full-extension scale, corresponding to approximately 60° of knee flexion; therefore, the findings should not be interpreted as evidence that this angle is superior to other testing positions. In addition, the FTSST and MVIC represent related but distinct constructs. The FTSST reflects dynamic, task-level rising performance, whereas MVIC reflects isolated isometric knee extensor force capacity under standardized testing conditions. This distinction may explain why smartphone-derived movement variables were strongly associated with MVIC but should not be considered direct substitutes for dynamometry.

From a biomechanical and human movement perspective, the sit-to-stand transition is a mechanically demanding task of daily living [28]. Successful execution requires sufficient lower-extremity joint torques to accelerate the body center of mass upward and forward against gravity [29,30,31]. The knee extensor muscles play an important role during the vertical lift-off and extension phases [5,32].

The stronger association of smartphone-estimated relative vertical power with MVIC may reflect its ability to capture task-level rising-phase performance, which is more mechanically relevant to knee extensor force generation than total task time alone. Unlike total FTSST time, which aggregates rising, sitting, and transition periods, smartphone-estimated relative vertical power incorporates temporal and displacement-related information from the concentric rising phase. This interpretation is also consistent with evidence that chair-rising power can provide additional information beyond chair-rising time when evaluating functional power across adulthood and older age [33].

The stronger performance of smartphone-estimated relative vertical power should also be interpreted in light of how this variable was derived. Unlike force-plate analysis or inverse dynamics, the smartphone application does not directly measure ground reaction force, mechanical work, or joint-level muscle power. Instead, the estimate combines temporal and displacement-related information within an empirically calibrated regression equation. Therefore, its clinical value lies in providing an accessible task-level movement estimate associated with knee extensor force capacity, rather than in directly quantifying mechanical power.

Vertical velocity also demonstrated a significant predictive relationship with knee extensor strength. In human movement science, the velocity of the center of mass during sit-to-stand is recognized as a determinant of dynamic balance and successful task completion [34,35]. Generating adequate vertical velocity is necessary to transition the body from a large and stable base of support, the chair, to a smaller and less stable base of support, the feet [14,36]. Older adults with reduced lower-limb strength may adopt slower rising strategies to maintain postural stability and reduce mechanical demand on weakened joints [37,38]. However, while velocity is informative, it remains inferior to smartphone-estimated relative vertical power as a predictive tool because it does not incorporate displacement-related and task-level power information from the app-derived equation.

The revised analyses intentionally excluded bioelectrical impedance-derived body composition variables from the main predictive models. This decision was made to avoid over-interpreting device-specific body composition estimates and to strengthen the transparency and reproducibility of the prediction models. The retained predictors were based on smartphone-derived kinematic variables and simple participant characteristics that can be obtained in community-based settings. Importantly, this revision sharpened the central interpretation of the study: the predictive value of the model is primarily attributable to the movement information captured during the sit-to-stand rising phase rather than to estimated body composition.

Although the apparent adjusted R^2^ of the final power-based model was high, this value represents in-sample explanatory performance and may overestimate prediction performance in new individuals. This concern is particularly relevant because the model included four predictors in a sample of 105 participants. The repeated 10-fold cross-validation results provide a more conservative estimate of expected predictive performance, with CV-adjusted R^2^ lower than the apparent adjusted R^2^. These findings suggest acceptable internal stability, but they also indicate that the model should be interpreted as an internally validated screening approach rather than as a fully generalizable prediction equation. External validation in an independent cohort remains necessary before broad clinical implementation.

The Bland–Altman findings further support cautious clinical interpretation. Although the mean bias was negligible and only a small proportion of observations fell outside the 95% limits of agreement, significant directional proportional bias was present. The model tended to overestimate MVIC among weaker individuals and underestimate MVIC among stronger individuals. This direction of bias is particularly important for screening because overestimation in weaker individuals may reduce sensitivity for identifying low strength. Some older adults with genuinely low knee extensor strength may be misclassified as having acceptable strength if a rigid smartphone-derived cut-off is applied. Therefore, smartphone-estimated relative vertical power should be used as a first-step screening, monitoring, or risk-stratification indicator rather than as a stand-alone diagnostic substitute for standardized dynamometry. Older adults with low, borderline, or clinically suspicious estimated values should undergo confirmatory knee extensor strength assessment using standardized dynamometry.

From a clinical perspective, the ±6.3 kg limits of agreement are relatively wide for precise individual-level MVIC estimation. Therefore, smartphone-estimated relative vertical power should not be used as a stand-alone substitute for standardized dynamometry. Its more appropriate role is as an accessible first-step screening or monitoring indicator in community settings, with confirmatory dynamometry recommended for individuals with low, borderline, or clinically suspicious estimated values.

Another methodological issue is that the app-derived equation uses femur length as a practical anthropometric proxy for vertical displacement rather than directly measuring greater-trochanter vertical excursion. Actual displacement during sit-to-stand can be influenced by chair height, hip flexion-extension range, trunk inclination, and individual rising strategy. This assumption may contribute to residual prediction error and partly explain individual-level disagreement between predicted and measured MVIC. Future studies should evaluate whether exported marker trajectories, force plates, instrumented-chair systems, or three-dimensional motion analysis improve the calibration and accuracy of smartphone-estimated relative vertical power.

### Limitations and Future Research Directions

Several limitations should be acknowledged. First, although the final power-based model showed acceptable internal stability after repeated 10-fold cross-validation, it has not yet been externally validated in an independent cohort. The use of convenience sampling from a single community-based region and the predominance of female participants may also limit generalizability to broader older-adult populations, particularly older men. Future studies should therefore validate the model in larger, independent, and more sex-balanced cohorts.

Second, smartphone-estimated relative vertical power was derived from a regression-based app algorithm rather than from direct force-plate, instrumented-chair, inverse-dynamics, or three-dimensional motion-analysis measurements. In addition, femur length was used as a practical anthropometric proxy for vertical displacement rather than as a direct measure of whole-body center-of-mass or greater-trochanter displacement. These methodological choices were appropriate for an accessible community-based assessment, but they may contribute to residual prediction error. Future studies comparing smartphone-derived estimates with laboratory-based kinetic and kinematic measurements would help refine model calibration.

Third, although the Bland–Altman analysis showed negligible group-level mean bias, the presence of proportional bias suggests that individual-level estimates should be interpreted cautiously. Smartphone-estimated relative vertical power may therefore be most suitable for community-based screening, monitoring, or risk stratification, with standardized dynamometry remaining the preferred method for precise strength quantification. Future studies should further evaluate clinically meaningful cut-off values and measurement reliability.

## 5. Conclusions

Consistent with the study hypothesis, smartphone-estimated relative vertical power was the strongest kinematic predictor of seated isometric knee extensor MVIC among the evaluated FTSST-derived variables. The final model, which included sex, age, femur length, and smartphone-estimated relative vertical power, showed the best predictive performance and acceptable internal stability after repeated 10-fold cross-validation. These findings suggest that smartphone-based analysis of the rising phase of the FTSST may provide clinically useful information beyond total task completion time. This approach may support accessible community-based screening, monitoring, and risk stratification of lower-limb strength in older adults, particularly in settings where standardized dynamometry is not readily available. It may help identify individuals who require confirmatory strength testing, closer follow-up, or targeted exercise intervention. Nevertheless, smartphone-estimated relative vertical power is a regression-derived estimate rather than a direct mechanical power measurement, and directional proportional bias was observed, with overestimation among weaker individuals and underestimation among stronger individuals. Therefore, this approach should not be used as a stand-alone diagnostic substitute for standardized dynamometry, and external validation in larger, independent, and more sex-balanced cohorts is required before broad clinical implementation or diagnostic cut-off development.

## Figures and Tables

**Table 1:** Participant demographic, anthropometric, BIA-derived body composition, functional, and smartphone-derived kinematic characteristics in the total sample and stratified by sex.

Variable	Total Sample (*n* = 105)	Women (*n* = 80)	Men (*n* = 25)
Sex, ***n*** (%)	25 men (23.8%)/ 80 women (76.2%)	80 (76.2%)	25 (23.8%)
Age (years)	68.19 ± 5.85	68.49 ± 6.01	67.24 ± 5.32
Height (cm)	153.64 ± 8.98	150.35 ± 6.89	164.16 ± 6.45
Body weight (kg)	53.39 ± 11.14	52.35 ± 10.65	56.73 ± 12.21
BMI (kg/m^2^)	22.63 ± 4.42	23.13 ± 4.38	21.04 ± 4.25
Body fat (%)	28.25 ± 11.99	31.12 ± 11.69	19.05 ± 7.60
Bone mass (kg)	2.05 ± 0.42	1.95 ± 0.37	2.39 ± 0.40
Skeletal muscle mass (kg)	36.02 ± 6.71	33.63 ± 4.02	43.64 ± 7.90
Skeletal muscle mass index (kg/m^2^)	15.20 ± 2.01	14.88 ± 1.53	16.23 ± 2.87
Femur length (cm)	34.19 ± 2.57	33.90 ± 2.48	35.14 ± 2.68
Calf circumference (cm)	32.49 ± 3.64	32.14 ± 3.65	33.62 ± 3.43
FTSST total time (s)	11.94 ± 2.93	12.40 ± 2.95	10.48 ± 2.36
Rising time (s)	0.95 ± 0.23	0.97 ± 0.24	0.89 ± 0.16
Vertical velocity (m/s)	0.38 ± 0.09	0.36 ± 0.09	0.42 ± 0.08
Smartphone-estimated relative vertical power (W/kg)	3.13 ± 1.24	2.81 ± 1.13	4.17 ± 1.00
MVIC of KE muscle strength (kg)	22.78 ± 6.15	20.81 ± 5.16	29.06 ± 4.69

Note: Values are presented as mean ± SD unless otherwise indicated. BMI = body mass index; BIA = bioelectrical impedance analysis; SMI = skeletal muscle mass index; FTSST = Five Times Sit-to-Stand Test; MVIC = maximum voluntary isometric contraction; KE = knee extensor. Body fat percentage, bone mass, skeletal muscle mass, and SMI were calculated directly from the raw BIA-related data fields.

**Table 2:** Pearson and Spearman correlations between knee extensor MVIC, non-BIA participant characteristics, and FTSST-derived variables.

Variable	Pearson r	95% CI for Pearson r	*p*-Value	Spearman ρ	*p*-Value
Sex	0.574	0.430 to 0.690	<0.001 ***	0.566	<0.001 ***
Age	−0.495	−0.627 to −0.335	<0.001 ***	−0.535	<0.001 ***
Body weight	0.249	0.060 to 0.421	0.010 *	—	—
Height	0.502	0.343 to 0.633	<0.001 ***	—	—
BMI	−0.025	−0.216 to 0.167	0.797	—	—
Femur length	0.451	0.284 to 0.592	<0.001 ***	0.403	<0.001 ***
Calf circumference	0.208	0.017 to 0.384	0.033 *	—	—
FTSST total time	−0.489	−0.622 to −0.328	<0.001 ***	−0.513	<0.001 ***
Rising time	−0.490	−0.623 to −0.329	<0.001 ***	−0.421	<0.001 ***
Vertical velocity	0.581	0.438 to 0.695	<0.001 ***	0.586	<0.001 ***
Smartphone-estimated relative vertical power	0.787	0.701 to 0.850	<0.001 ***	0.749	<0.001 ***

Note: Correlation analyses were exploratory and were used to describe bivariate relationships with MVIC. Benjamini–Hochberg false-discovery rate correction was applied to account for multiple bivariate tests; all statistically significant correlations shown here remained significant after correction. Body weight and calf circumference were retained as descriptive anthropometric variables and were examined in sensitivity analysis, whereas confirmatory interpretation was based on the prespecified adjusted regression models. r = Pearson correlation coefficient; BMI = body mass index; FTSST = Five Times Sit-to-Stand Test; MVIC = maximum voluntary isometric contraction. * *p* < 0.05, *** *p* < 0.001.

**Table 3:** Multiple regression models predicting knee extensor MVIC after excluding bioelectrical impedance-derived body composition variables.

Model and Predictors	B	95% CI for B	SE	β	*p*-Value	VIF	Adjusted R^2^	SEE	AIC	BIC
Power-based model							0.716	3.276	254.052	267.322
Constant	15.090	2.150 to 28.030	6.522	—	0.023 *	—				
Sex	4.192	2.477 to 5.907	0.865	0.292	<0.001 ***	1.327				
Age	−0.212	−0.339 to −0.086	0.064	−0.202	0.001 **	1.349				
Femur length	0.397	0.128 to 0.665	0.135	0.166	0.004 **	1.175				
Smartphone-estimated relative vertical power	2.423	1.717 to 3.129	0.356	0.488	<0.001 ***	1.881				
Velocity-based model							0.658	3.596	273.647	286.916
Constant	18.866	4.744 to 32.988	7.118	—	0.009 **	—				
Sex	6.001	4.285 to 7.718	0.865	0.418	<0.001 ***	1.102				
Age	−0.337	−0.464 to −0.210	0.064	−0.321	<0.001 ***	1.128				
Femur length	0.525	0.237 to 0.813	0.145	0.220	<0.001 ***	1.124				
Vertical velocity	19.762	11.297 to 28.227	4.267	0.298	<0.001 ***	1.256				
Rising-time model							0.631	3.732	281.433	294.703
Constant	29.777	15.497 to 44.057	7.197	—	<0.001 ***	—				
Sex	6.593	4.849 to 8.338	0.879	0.459	<0.001 ***	1.058				
Age	−0.330	−0.466 to −0.194	0.069	−0.314	<0.001 ***	1.204				
Femur length	0.584	0.288 to 0.880	0.149	0.245	<0.001 ***	1.101				
Rising time	−6.355	−9.883 to −2.827	1.778	−0.237	<0.001 ***	1.243				
FTSST total-time model							0.623	3.774	283.803	297.073
Constant	31.002	16.438 to 45.566	7.342	—	<0.001 ***	—				
Sex	6.183	4.372 to 7.993	0.913	0.431	<0.001 ***	1.113				
Age	−0.367	−0.499 to −0.235	0.067	−0.350	<0.001 ***	1.108				
Femur length	0.603	0.305 to 0.901	0.150	0.253	<0.001 ***	1.093				
FTSST total time	−0.443	−0.718 to −0.169	0.138	−0.211	0.002 **	1.197				

Note: All models were adjusted for sex, age, and femur length. Sex was coded as 0 = female and 1 = male. Unstandardized regression coefficients are presented with 95% confidence intervals. The four candidate kinematic models were non-nested because each model contained a different FTSST-derived predictor; therefore, they were compared using adjusted R^2^, SEE, AIC, and BIC rather than direct partial F-tests. Nested model comparison was performed between the demographics-only model and the power-based model using ΔR^2^ and partial F-change. VIF was used to assess multicollinearity, with values greater than 10 prespecified as indicating problematic multicollinearity. MVIC = maximum voluntary isometric contraction; FTSST = Five Times Sit-to-Stand Test; SEE = standard error of the estimate; AIC = Akaike information criterion; BIC = Bayesian information criterion; VIF = variance inflation factor. * *p* < 0.05, ** *p* < 0.01, *** *p* < 0.001.

**Table 4:** Incremental value, internal validation, and sensitivity analysis of the final power-based model.

Analysis	Result
Demographics-only baseline model R^2^	0.600
Demographics-only baseline adjusted R^2^	0.588
Final power-based model R^2^	0.727
Final power-based model adjusted R^2^	0.716
ΔR^2^ after adding smartphone-estimated relative vertical power	0.127
Partial R^2^ for smartphone-estimated relative vertical power	0.318
Partial F-change	F (1, 100) = 46.371, *p* < 0.001
Apparent RMSE	3.197 kg
Apparent MAE	2.609 kg
Cross-validated R^2^	0.701
Cross-validated adjusted R^2^	0.689
Cross-validated RMSE	3.343 kg
Cross-validated MAE	2.739 kg
Sex × smartphone-estimated relative vertical power interaction	B = −0.145, 95% CI = −1.636 to 1.346, *p* = 0.847
Sensitivity model adjusted R^2^ after adding body weight and calf circumference	0.721
Smartphone-estimated relative vertical power in sensitivity model	B = 2.437, 95% CI = 1.736 to 3.138, *p* < 0.001
Body weight in sensitivity model	B = 0.035, 95% CI = −0.040 to 0.110, *p* = 0.360
Calf circumference in sensitivity model	B = 0.085, 95% CI = −0.144 to 0.315, *p* = 0.463

Note: Internal validation was performed using repeated 10-fold cross-validation with 100 repetitions. RMSE = root mean square error; MAE = mean absolute error.

## Data Availability

The datasets generated and analyzed during the current study are not publicly distributed to protect the privacy and confidentiality of the older adult participants, in strict compliance with the ethical protocols. However, anonymized data supporting the findings of this study are available from the corresponding author (W.T.) upon reasonable academic request and subject to appropriate ethical clearance.

## References

[1] Clark B.C. (2019). Neuromuscular Changes with Aging and Sarcopenia. J. Frailty Aging.

[2] Pabla P., Jones E.J., Piasecki M., Phillips B.E. (2024). Skeletal muscle dysfunction with advancing age. Clin. Sci..

[3] Cruz-Jentoft A.J., Bahat G., Bauer J., Boirie Y., Bruyère O., Cederholm T., Cooper C., Landi F., Rolland Y., Sayer A.A. (2019). Sarcopenia: Revised European consensus on definition and diagnosis. Age Ageing.

[4] Seko T., Akasaka H., Koyama M., Himuro N., Saitoh S., Ogawa S., Miura S., Mori M., Ohnishi H. (2024). The Contributions of Knee Extension Strength and Hand Grip Strength to Factors Relevant to Physical Frailty: The Tanno-Sobetsu Study. Geriatrics.

[5] Tapanya W., Sangkarit N., Amput P., Konsanit S. (2024). Lower extremity muscle strength equation of older adults assessed by Five Time Sit to Stand Test (FTSST). Hong Kong Physiother. J..

[6] Cosentino S., Kasai R., Gu Z., Sessa S., Kawakami Y., Takanishi A. Knee extensor muscular activity estimation during different walking patterns: Flat normal and brisk walking, stair climbing. Proceedings of the 2018 40th Annual International Conference of the IEEE Engineering in Medicine and Biology Society (EMBC).

[7] Kojima N., Kim H., Saito K., Yoshida H., Yoshida Y., Hirano H., Obuchi S., Shimada H., Suzuki T. (2014). Association of knee-extension strength with instrumental activities of daily living in community-dwelling older adults. Geriatr. Gerontol. Int..

[8] Borges V., Silva N., Malta A.C., Xavier N.C., Santana Bernardes L.E. (2017). Falls, Muscle Strength, and Functional Abilities in Community-Dwelling Elderly Women. Arch. Phys. Med. Rehabil..

[9] Hong J.-S., Ko J.-B., Ju M.-M., Lee B.-K., Park D.-S., Lee S.-H. (2025). The Reliability and Validity of an Isometric Knee Strength Measurement Device in Older Adult Individuals. Sensors.

[10] Stark T., Walker B., Phillips J.K., Fejer R., Beck R. (2011). Hand-held Dynamometry Correlation With the Gold Standard Isokinetic Dynamometry: A Systematic Review. Pmr.

[11] Takeno K., Katch V.L., Norte G.E., Ingersoll C.D. (2025). Validity and reliability of a novel portable tension-gauge dynamometer for isometric and isotonic seated knee extension strength measurement. Knee.

[12] Park T.S., Shin M.-J. (2024). Comprehensive Assessment of Lower Limb Function and Muscle Strength in Sarcopenia: Insights from the Sit-to-Stand Test. Ann. Geriatr. Med. Res..

[13] Sadeh S., Gobert D., Shen K.-H., Foroughi F., Hsiao H.-Y. (2023). Biomechanical and neuromuscular control characteristics of sit-to-stand transfer in young and older adults: A systematic review with implications for balance regulation mechanisms. Clin. Biomech..

[14] Ahn N., Lewis C.L., Kipp K. (2025). Joint-Specific Contributions to Vertical and Horizontal Center-of-Mass Velocity During a Sit-to-Stand Task Depend on Age. J. Appl. Biomech..

[15] Crockett K., Ardell K., Hermanson M., Penner A., Lanovaz J., Farthing J., Arnold C. (2013). The Relationship of Knee-Extensor Strength and Rate of Torque Development to Sit-to-Stand Performance in Older Adults. Physiother. Can..

[16] Gluchowski A., Phillips S.M. (2025). Antifrail: Why Muscle (Power) Matters in Aging. ACSM’s Health Fit. J..

[17] Reid K.F., Fielding R.A. (2012). Skeletal Muscle Power: A Critical Determinant of Physical Functioning in Older Adults. Exerc. Sport. Sci. Rev..

[18] Carlson B., Carlson B. (2022). Chapter 8—The Aging of Muscle. Muscle Biology.

[19] Dowling P., Gargan S., Swandulla D., Ohlendieck K. (2023). Fiber-Type Shifting in Sarcopenia of Old Age: Proteomic Profiling of the Contractile Apparatus of Skeletal Muscles. Int. J. Mol. Sci..

[20] Tateoka K., Tsuji T., Shoji T., Tokunaga S., Okura T. (2023). Relationship between Acceleration in a Sit-To-Stand Movement and Physical Function in Older Adults. Geriatrics.

[21] Ruiz-Cárdenas J.D., Montemurro A., Martínez-García M.D.M., Rodríguez-Juan J.J. (2023). Sit-to-Stand Video Analysis-Based App for Diagnosing Sarcopenia and Its Relationship With Health-Related Risk Factors and Frailty in Community-Dwelling Older Adults: Diagnostic Accuracy Study. J. Med. Internet Res..

[22] Chan L.C., Yan J., Zhang Y.C., Jiang T., Zhang A.Y., Li H.H.T., So B., Huang W., Zheng Y., Chan P.K. (2026). Smartphone-derived joint angular velocities in sit-to-stand motion provide a spatiotemporal marker for symptomatic knee osteoarthritis. Commun. Med..

[23] Ruiz-Cárdenas J.D., Rodríguez-Juan J.J., Smart R.R., Jakobi J.M., Jones G.R. (2018). Validity and reliability of an iPhone App to assess time, velocity and leg power during a sit-to-stand functional performance test. Gait Posture.

[24] Tapanya W., Sangkarit N., Manoy P., Konsanit S. (2024). Modified Squat Test for Predicting Knee Muscle Strength in Older Adults. Ann. Geriatr. Med. Res..

[25] Tapanya W., Maharan S., Amput P., Sangkarit N., Suwannakul B. (2023). The Influence of Knee Extensor and Ankle Plantar Flexor Strength on Single-Leg Standing Balance in Older Women. J. Funct. Morphol. Kinesiol..

[26] Collins G.S., Reitsma J.B., Altman D.G., Moons K.G. (2015). Transparent reporting of a multivariable prediction model for individual prognosis or diagnosis (TRIPOD): The TRIPOD statement. J. Br. Surg..

[27] Bland J.M., Altman D.G. (1986). Statistical methods for assessing agreement between two methods of clinical measurement. Lancet.

[28] Yang J., Ozsoy B., Scataglini S., Paul G. (2019). Chapter 27—Physics-Based Sit-to-Stand Three-Dimensional Motion Prediction Considering Seat Pan Contact. DHM and Posturography.

[29] Yoshioka S., Nagano A., Himeno R., Fukashiro S. (2007). Computation of the kinematics and the minimum peak joint moments of sit-to-stand movements. Biomed. Eng. Online.

[30] Caruthers E.J., Thompson J.A., Chaudhari A.M.W., Schmitt L.C., Best T.M., Saul K.R., Siston R.A. (2016). Muscle Forces and Their Contributions to Vertical and Horizontal Acceleration of the Center of Mass During Sit-to-Stand Transfer in Young, Healthy Adults. J. Appl. Biomech..

[31] Soni V., Vaz A. (2024). Dynamics of sit-to-stand and stand-to-sit motions based on the trajectory control of the centre of mass of the body: A bond graph approach. Comput. Biol. Med..

[32] O’Keeffe C., Gill C., Etzelmueller M., Taylor C., Hablani S., Reilly R.B., Fleming N. (2023). Multimodal analysis of the biomechanical impact of knee angle on the Sit-to-Stand transition. Gait Posture.

[33] Rittweger J., Gollasch M., Dietzel R., Armbrecht G. (2025). Chair-Rising Power as Digital Biomarker: Validation against Jumping Power and Chair-Rising Time in Adults Aged 32–92 Years. Digit. Biomark..

[34] Mourey F., Grishin A., d’Athis P., Pozzo T., Stapley P. (2000). Standing Up From a Chair as a Dynamic Equilibrium Task: A Comparison Between Young and Elderly Subjects. J. Gerontol. Ser. A.

[35] Yamada T., Demura S., Takahashi K. (2013). Center of gravity transfer velocity during sit-to-stand is closely related to physical functions regarding fall experience of the elderly living in community dwelling. Health.

[36] Schenkman M., Berger R.A., Riley P.O., Mann R.W., Hodge W.A. (1990). Whole-Body Movements During Rising to Standing from Sitting. Phys. Ther..

[37] Wrucke D.J., Kuplic A., Adam M., Hunter S.K., Sundberg C.W. (2022). Mechanisms For The Age-related Loss In Power Of The Knee Extensors In Men And Women: 1639. Med. Sci. Sports Exerc..

[38] Ma G., Cao C., Zhang T., Zheng H., Song Q., Zhang C., Sun W., Wang J. (2023). The Lower Limb Stiffness, Moments, and Work Mode During Stair Descent Among the Older Adults. Am. J. Phys. Med. Rehabil..

